# Relation between baseline plaque features and subsequent coronary artery remodeling determined by optical coherence tomography and intravascular ultrasound

**DOI:** 10.18632/oncotarget.13959

**Published:** 2016-12-15

**Authors:** Zulong Xie, Nana Dong, Rong Sun, Xinxin Liu, Xia Gu, Yong Sun, Hongwei Du, Jiannan Dai, Youbin Liu, Jingbo Hou, Jinwei Tian, Bo Yu

**Affiliations:** ^1^ Department of Cardiology, The Second Affiliated Hospital of Harbin Medical University, Harbin, China; ^2^ Department of Cardiology, The Second Affiliated Hospital of Chongqing Medical University, Chongqing, China; ^3^ The Key Laboratory of Myocardial Ischemia, Chinese Ministry of Education, Harbin, China; ^4^ Department of Cardiology, Heilongjiang Provincial Hospital, Harbin, China

**Keywords:** atherosclerosis, plaque characteristics, coronary artery remodeling, optical coherence tomography, intravascular ultrasound

## Abstract

Atherosclerosis often leads to myocardial infarction and stroke. We examined the influence of baseline plaque characteristics on subsequent vascular remodeling in response to changes in plaque size. Using optical coherence tomography (OCT) and intravascular ultrasound (IVUS), we examined 213 plaques from 138 patients with acute coronary syndrome at baseline and repeated IVUS at the 12-month follow-up. The change in external elastic membrane (EEM) area for each 1 mm2 change in plaque area (i.e., the slope of the regression line) was calculated as a measure of vascular remodeling capacity. In plaques with static positive remodeling, the slope was smaller than in plaques without static positive remodeling. In addition, the slope of the regression line for lesions with a large plaque burden was much smaller than that for lesions with a small plaque burden. Multivariate linear regression analysis showed that diabetes, calcification and static positive remodeling were inversely and independently associated with the level of change in EEM area/change in plaque area. Lesions with a large plaque burden, calcifications or static positive remodeling had less remodeling capacity, and calcification and static positive remodeling were independent predictors of reduced subsequent remodeling. Therefore, calcifications and static positive remodeling could be used as morphological biomarkers to predict decreased subsequent arterial remodeling.

## INTRODUCTION

Atherosclerosis is the main underlying cause of myocardial infarction and stroke and accounts for greater than one-half of all deaths worldwide. Arterial remodeling in response to plaque accumulation has been widely recognized as a mechanism for lumen preservation during coronary atherosclerosis development [1–4]. Intravascular ultrasound (IVUS) is an established imaging technology that can be used to evaluate vascular structure geometry and study coronary arterial remodeling *in vivo* [5–8]. In the static method of assessment, remodeling of individual lesions is determined by comparing the external elastic membrane (EEM) area of the lesion site to the mean EEM area of the proximal and distal reference sites [5, 9]. However, the likelihood that the reference site has also undergone remodeling makes this method somewhat unreliable. Direct evidence of vascular remodeling requires the serial method of assessment in which the lesion EEM area is measured and compared over two or more time points [5]. Multiple prior cross-sectional IVUS studies have reported an association between vulnerable plaque characteristics and static vascular remodeling [9, 10]; yet, further research is needed to determine the influence of plaque characteristics on serial vascular remodeling.

Intracoronary optical coherence tomography (OCT) is an emerging technology that enables cross-sectional vascular imaging with approximately 10-20 μm resolution. The relatively high resolution of OCT makes it a powerful tool for both quantitative plaque analysis and qualitative plaque characterization [11, 12]. OCT can discriminate between early and advanced-stage lesions more accurately than coronary computed tomographic angiography and IVUS [13]. We hypothesize that the stage and underlying morphologic characteristics of atherosclerotic lesions influence the vascular response to changes in plaque size. In this study, we analyzed baseline OCT and serial IVUS images to determine the influence of baseline plaque characteristics on serial coronary remodeling in patients with acute coronary syndrome (ACS).

## RESULTS

Patient clinical characteristics and concomitant medications during follow-up are summarized in Table 1. Most patients were discharged on a statin and dual antiplatelet therapy and continued this treatment during the subsequent 12-month follow-up period. Lipid profiles improved on follow-up measurement, compared to baseline (Table 2).

**Table 1:** Patient characteristics

Variables	Values (*n* total = 138)
Age (years)	56.5 ± 9.3
Male, *n* (%)	96 (69)
Smoking, *n* (%)	80 (58)
Diabetes mellitus, *n* (%)	55 (40)
Hypertension, *n* (%)	79 (57)
Hyperlipidemia, *n* (%)	65 (47)
STEMI, *n* (%)	33 (24)
NSTE-ACS, *n* (%)	105 (76)
Concomitant medications	
Statins, *n* (%)	130 (94)
Beta-blockers, *n* (%)	82 (59)
ACEI or ARB, *n* (%)	64 (46)
CCB, *n* (%)	39 (28)
Aspirin, *n* (%)	137 (99)
Clopidogrel, *n* (%)	135 (98)

Values are presented as the mean ± SD or n (%). ACEI, angiotensin converting enzyme inhibitor; ARB, angiotensin receptor blocker; CCB, calcium channel blocker; NSTE-ACS, non-ST-segment elevation acute coronary syndromes.

**Table 2:** Laboratory values

Variables	Baseline	Follow-up	*P*-value
TC, mg/dl	189.1 ± 41.8	147.3 ± 39.1	<0.001
LDL-C, mg/dl	101.3 ± 30.9	75.4 ± 28.6	<0.001
HDL-C, mg/dl	49.5 ± 12.4	45.6 ± 13.1	<0.001
TG, mg/dl	188.7 ± 95.7	136.4 ± 100.1	<0.001
hs-CRP, mg/dl	2.66 (0.94, 6.49)	0.84 (0.52, 1.67)	<0.001

Categorical data are presented as n (%). Continuous data are presented as the mean ± SD or as median (25th, 75th). HDL-C, high-density lipoprotein cholesterol; hs-CRP, high-sensitivity C-reactive protein; LDL-C, low-density lipoprotein cholesterol; TC, total cholesterol; TG, triglyceride.

### Baseline OCT-derived and IVUS-derived plaque features

The baseline angiographic and OCT-derived plaques features are presented in Table 3. Plaques were located most frequently in the right coronary artery (RCA), followed by the left anterior descending artery (LAD), and least frequently in the left circumflex artery (LCX). Sixty-one of the lesions (29%) were identified as fibrous plaques. Forty-five of the lesions (21%) were diagnosed as OCT-defined TCFA (thin-cap fibroatheroma). The incidence of microvessels, cholesterol crystals, calcifications, and macrophages was 43, 26, 39, and 49%, respectively. The IVUS findings are presented in Table 4. The prevalence of positive static remodeling, spotty calcifications, and attenuated plaques was 26, 28, and 25%, respectively. Changes in mean EEM, lumen, and plaque area from baseline to the 12-month follow-up evaluation as assessed using serial IVUS, are listed in Table 5.

**Table 3:** Baseline angiographic and OCT-derived plaque characteristics

	Values (*n* = 213)
Lesion location	
RCA, *n* (%)	93 (44)
LAD, *n* (%)	79 (37)
LCX, *n* (%)	41 (19)
QCA findings	
MLD, mm	1.86 ± 0.53
RLD, mm	3.09 ± 0.67
DS, %	40.3 ± 10.5
Lesion length, mm	10.8 ± 4.5
OCT-derived plaque characteristics	
Lipid length, mm	12.1 ± 7.3
Lipid arc, °	192.4 ± 62.9
Fibrous cap thickness, μm	117.2 ± 74.9
Fibrous plaque, *n* (%)	61 (29)
TCFA, *n* (%)	45 (21)
Microvessel, *n* (%)	91 (43)
Cholesterol crystal, *n* (%)	56 (26)
Calcification, *n* (%)	82(39)
Macrophage, *n* (%)	104 (49)

Categorical data are presented as n (%). Continuous data are presented as the mean ± SD. MLD, minimum lumen diameter; RLD, reference lumen diameter; DS, diameter stenosis.

**Table 4:** Baseline IVUS-derived plaque characteristics

	Values (*n* = 213)
Lesion site	
EEM area, mm^2^	14.0 ± 5.05
Lumen area, mm^2^	5.06 ± 2.10
Plaque area, mm^2^	8.93 ± 3.84
Plaque burden, %	62.8 ± 10.4
Static remodeling index	0.97 ± 0.14
Static positive remodeling, *n* (%)	56 (26)
Spotty calcification, *n* (%)	60 (28)
Attenuated plaque, *n* (%)	53 (25)
Proximal reference site	
EEM area, mm^2^	15.8 ± 7.0
Lumen area, mm^2^	8.17 ± 3.13
Distal reference site	
EEM area, mm^2^	13.2 ± 4.9
Lumen area, mm^2^	7.50 ± 3.11

Categorical data are presented as n (%). Continuous data are presented as the mean ± SD.

**Table 5:** Mean EEM, lumen, and plaque areas

Variables	Baseline	Follow-up	Change over time	*P*-value
Mean EEM area, mm^2^	13.9 ± 4.7	13.5 ± 4.7	−0.37 ± 1.76	0.002
Mean lumen area, mm^2^	6.42 ± 2.32	6.29 ± 2.39	−0.14 ± 1.32	0.133
Mean plaque area, mm^2^	7.46 ± 3.06	7.23 ± 3.08	−0.23 ± 1.02	0.001

Values are presented as the mean ± SD.

### Relation between OCT-derived plaque features and vascular remodeling

**Figure 1:**
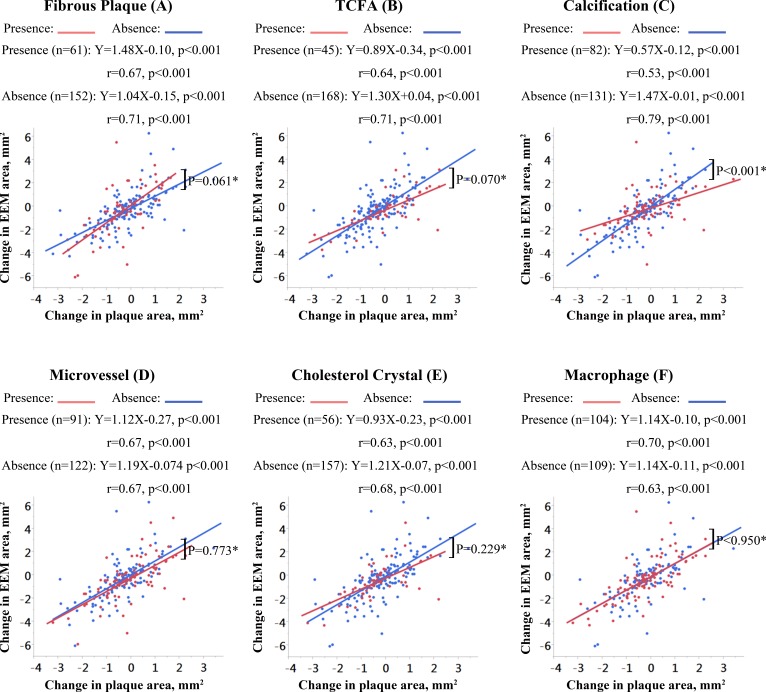
Correlations of change in EEM area with change in plaque area according to OCT-derived plaque characteristics The slopes, y-intercepts, and correlation coefficients are shown for all linear regression lines. *Comparison of the slopes of the regression lines.

As shown in Figure 1, the correlation between changes in plaque and EEM areas was positive and statistically significant for all subgroups stratified by baseline OCT-derived plaque characteristics. The change in EEM area for each 1 mm^2^ change in plaque area (i.e., the slope of the regression line) was greater for fibrous plaques compared to non-fibrous plaques but did not reach statistical significance (slopes = 1.48 vs. 1.04 mm^2^, p = 0.061; Figure 1A). There was a trend towards a smaller slope of the regression line in plaques with TCFA compared to plaques without TCFA (slopes = 0.89 vs. 1.30 mm^2^, *p* = 0.070; Figure 1B). Of note, there was a statistically significant difference in the regression line slope between lesions with and without calcification, with calcified lesions showing less change in EEM area relative to change in plaque area (slopes: 0.57 vs. 1.47 mm^2^, *p* < 0.001; Figure 1C). The slopes of the regression lines were not significantly affected by the presence or absence of microvessels (*p* = 0.773; Figure 1D), cholesterol crystals (*p* = 0.229; Figure 1E), or macrophages (*p* = 0.950; Figure 1F).

### Relation between IVUS-derived plaque features and vascular remodeling

**Figure 2:**
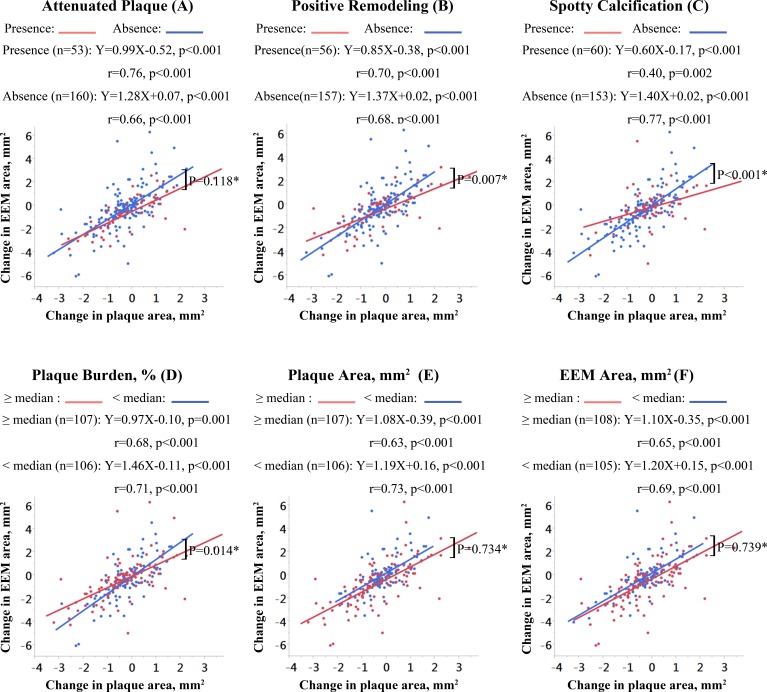
Correlations of change in EEM area with change in plaque area according to IVUS-derived plaque characteristics The slopes, y-intercepts, and correlation coefficients are shown for all linear regression lines. N/A, not available; *comparison of the slopes of the regression lines.

Across all subgroups stratified by IVUS-derived plaque characteristics, there was a positive correlation between changes in plaque and EEM areas (all *P* values < 0.05; Figure 2). Compared with non-attenuated plaques, the regression line for attenuated plaques was flatter but the difference failed to reach statistical significance (*p* = 0.118; Figure 2A). In plaques with static positive remodeling, the change in EEM area for each 1 mm^2^ change in plaque area was significantly smaller compared to plaques without static positive remodeling (slopes = 0.85 vs. 1.37 mm^2^, *p* = 0.007; Figure 2B). The slope of the regression line for plaques with spotty calcifications was significantly flatter than the slope for plaques without spotty calcifications (slopes = 0.60 vs. 1.40 mm2, *p* < 0.001; Figure 2C). The slope of the regression line for lesions with a larger plaque burden (≥ 64%; median value of the baseline plaque burden) was less steep than the slope for lesions with smaller plaque burden (< 64%; slopes = 0.97 vs. 1.46 mm2, *p* = 0.014; Figure 2D). The baseline plaque area or EEM area did not significantly affect the slopes of the regression line (*p* = 0.734, Figure 2E; *p* = 0.739, Figure 2F).

### Predictors for arterial remodeling capacity

By multivariate linear regression analysis, with the level of change in EEM area/change in plaque area as the dependent variable, the presence of diabetes mellitus (β = -0.704, *p* = 0.004), calcification (β = -0.601, *p* = 0.008), and static positive remodeling (β = -0.508, *p* = 0.049), were independently and negatively associated with arterial remodeling capacity (Table 6). Medications, other traditional coronary risk factors and plaque characteristics were not correlated with the level of change in EEM area/change in plaque area.

**Table 6:** Multivariate linear regression analysis of potential predictors for arterial remodeling capacity

Variable	β	95% CI	*P*-value
Age	−0.436	−0.891 – 0.020	0.061
Male	0.362	−0.141 – 0.865	0.158
Hyperlipidemia	0.451	−0.119 – 1.020	0.121
Smoking	−0.301	−0.788 – 0.187	0.227
Diabetes mellitus	−0.704	−1.181 – -0.228	**0.004**
Hypertension	−0.1	−0.742 – 0.543	0.761
ACEI/ARB	−0.056	−0.578 – 0.465	0.833
Beta-blockers	−0.317	−0.768 – 0.134	0.168
CCB	0.331	−0.290 – 0.952	0.296
Statin	−0.615	−1.891 – 0.661	0.345
Baseline LDL-C	−0.342	−0.893 – 0.210	0.225
Baseline triglycerides	−0.177	−0.704 – 0.350	0.511
Baseline hs-CRP	−0.009	−0.425 – 0.408	0.908
Fibrous plaque	0.071	−0.464 – 0.606	0.795
TCFA	−0.149	−0.702 – 0.403	0.596
Calcification	−0.601	−1.044 – -0.157	**0.008**
Mircrovessel	−0.17	−0.603 – 0.263	0.443
Cholesterol crystal	−0.014	−0.603 – 0.574	0.962
Macrophage	0.392	−0.047 – 0.831	0.08
Attenuated plaque	−0.019	−0.541 – 0.504	0.944
Positive remodeling	−0.508	−1.018 – 0.002	**0.049**
Plaque burden	−0.106	−0.587 – 0.374	0.665
Plaque area	−0.005	−0.714 – 0.704	0.989
EEM area	−0.373	−1.026 – 0.281	0.264

*The level of change in EEM area/change in plaque area represents the arterial remodeling capacity of each plaque.

## DISCUSSION

To the best of our knowledge, this is the first *in vivo* human study to systematically investigate the relationship between baseline plaque characteristics and serial vascular remodeling. The capacity for coronary arteries to remodel in response to changes in plaque size remains relatively robust in the presence of fibrous plaques and lesions with a small plaque burden. However, lesions with TCFA, calcifications, or statically assessed positive remodeling exhibit less serial remodeling capacity. As the ability of vessels to remodel varies when different plaque subtypes are present, our findings add a layer of complexity to the interpretation of studies investigating atherosclerotic progression and regression. Although additional studies are needed to further confirm this hypothesis, our results provide new insight into arterial remodeling capacity, and may lead to the development of new therapeutic strategies for the control of atherosclerosis.

### Coronary arterial remodeling

Expansive coronary arterial remodeling might prevent initial plaque buildup from compromising the luminal area [3], and in some cases, overcompensation can lead to an increase in lumen area [6]. Many prior studies have suggested that expansively-remodeled vessels harbor an increased amount of lipid and inflammatory material, making these lesions more vulnerable to rupture, thereby causing clinical events [6, 9, 10, 14]. The magnitude of static plaque remodeling has been shown to inversely correlate with the fibrotic composition of a plaque [10], and plaques with positive static remodeling have been associated with larger lipid core size, greater macrophage infiltration, thinner fibrous caps, and more frequent plaque rupture [9, 14]. It is not clear, however, whether pre-existing, statically-assessed remodeling influences the capacity for subsequent serially-assessed remodeling. Serial IVUS assessment of vessel dimensions can provide direct evidence of vascular remodeling [5–8, 15]. In the current study, we demonstrated that pre-existing arterial remodeling was independently associated with decreased capacity for arterial remodeling. Indeed, it seems logical for positively-remodeled plaques to have less capacity to outwardly remodel and accommodate additional plaque growth. This concept may support our finding from a previous study in which no significant difference in remodeling index was found between ruptured culprit plaques and ruptured non-culprit plaques or TCFA, despite the former having a larger plaque burden [16]. Additionally, the capacity for subsequent coronary remodeling may be further hindered in positively remodeled plaques by thinning of the arterial media and arterial damage [1, 14], which may limit the ability for a vessel to return to its pre-atherosclerotic condition despite plaque regression.

### Plaque burden and subsequent arterial remodeling

According to the analyses by Glagov *et al*. [2], expansive remodeling maintains luminal dimensions during early atherosclerosis until the burden of atheroma reaches 40%. This finding has been corroborated by early *in vivo* IVUS studies using the static method of assessment [3, 17]. In sharp contrast, IVUS studies using the serial method of arterial remodeling assessment have shown that vessel enlargement is not related to the extent of atheroma burden, as lesions with a > 40% plaque burden continue to develop compensatory vessel enlargement [18]. This threshold of a 40% plaque burden was derived from pathologic studies and therefore may not be directly applicable to *in vivo* coronary imaging [2]. In the present study, we used the median plaque burden in our study sample (64%) as a threshold value to discriminate between lesions with large versus small plaque burden. Although there was a strong positive correlation between changes in plaque and vessel EEM sizes for lesions with small and large plaque burdens, lesions with a larger plaque burden showed less capacity for arterial remodeling in response to changes in plaque area.

### Arterial remodeling during the early and advanced stages of atherosclerosis

OCT-defined fibrous plaque has been validated against histology as an early stage atherosclerotic lesion [13]. Interestingly, in the current study, the degree of change in vessel wall size in response to change in plaque area was larger in OCT-defined fibrous plaques compared to non-fibrous plaques. This suggests that arterial remodeling capacity remains robust in early-stage atherosclerotic plaques. In contrast, TCFA represent more advanced stage atherosclerotic lesions. In our study, lesions with TCFA showed reduced remodeling capacity and less preservation of the lumen area in response to a change in plaque area when compared to lesions without TCFA. Lesions with calcifications also showed less capacity for vascular remodeling in response to changes in plaque size compared with lesions without calcification. The presence of calcifications was independently associated with reduced subsequent arterial remodeling. This finding may relate to the established correlation between vascular calcification severity and arterial stiffness [19]. Taken together, these findings suggest that arterial remodeling capacity in response to change in plaque size remains robust in early-stage atherosclerotic plaques and comparatively diminished in more advanced-stage lesions.

Although anti-atherosclerotic therapies can modify plaque characteristics over time [20], baseline plaque characteristics remained independently predictive of future clinical events [21–23]. Previous studies have demonstrated that the presence of TCFA with a large plaque burden was associated with increased risk for future major adverse clinical events [21, 22]. Coronary calcium scores have also been shown to be predictive of future coronary heart disease-related events [23]. Although early-stage atherosclerotic plaques may be entirely reversible, more advanced plaques exhibit resistance to lipid-lowering therapy [24]. Similarly, in the current study, we found that more advanced-stage plaques such as TCFA and lesions with calcifications showed reduced remodeling capacity, and therefore a greater risk for ischemia with plaque size progression. As such, early treatment of patients at risk for coronary artery disease, as recommended in the 2013 guidelines from the American Heart Association and American College of Cardiology, may be particularly important in preventing the progression of early-stage atherosclerosis to more treatment-refractory advanced stage disease.

Several limitations of this study should be acknowledged. First, the retrospective nature of this study implies that the decision to use OCT and IVUS was operator- and situation-dependent, thus introducing selection bias. A prospective study would help validate the findings of this study. Second, our findings may not be applicable to all lesions, as we used plaque-specific inclusion and exclusion criteria. Only non-culprit lesions with mild to moderate stenosis were included in this study; therefore, the results cannot be directly generalized to ACS culprit lesions or lesions with severe stenosis. Third, both time-domain and frequency-domain OCT systems were used in the current study. Fourth, due to the limited penetration depth of OCT, the micro vessels in the deep positions of plaque might have been underestimated. Fifth, the definition of macrophage infiltration, albeit widely accepted and used in other OCT studies [25], has not been validated against histologic findings. Finally, serum cholesterol levels were well-controlled in this study, and our findings may not represent the true natural history of arterial remodeling in untreated patients.

In conclusion, we found that the capacity for coronary artery remodeling in response to plaque progression or regression varies among lesions with different baseline characteristics. Lesions with IVUS and OCT-identified features associated with early-stage atherosclerosis showed a more robust capacity for arterial remodeling compared to lesions with advanced-stage atherosclerotic features. Calcifications and static positive remodeling may serve as morphological biomarkers to predict decreased subsequent arterial remodeling. Future prospective in vivo studies are warranted to validate the findings from this retrospective study.

## MATERIALS AND METHODS

### Study population

Between November 2009 and November 2013, 167 ACS patients underwent OCT and IVUS examinations of non-culprit lesions after percutaneous coronary intervention to the culprit lesion. All patients subsequently underwent repeat IVUS imaging at the 12-month follow-up evaluation. All angiogram, IVUS, and OCT images were stored digitally for detailed analysis. The study protocol was approved by the Ethics Committee of the Second Affiliated Hospital of Harbin Medical University (Harbin, Heilongjiang, China), and all patients provided written informed consent.

Patients with unstable angina, non-ST segment elevation myocardial infarction (NSTEMI), or ST segment elevation myocardial infarction (STEMI) were enrolled. Unstable angina was defined as rest, new-onset, or accelerating angina. NSTEMI was defined as ischemic symptoms with elevated cardiac markers (troponin T/I or creatine kinase-MB) but without ST-segment elevation on a 12-lead electrocardiogram. STEMI was defined as continuous chest pain > 30 min, ST-segment elevation > 0.1 mV in ≥ 2 contiguous leads or new left bundle-branch block on electrocardiogram, and elevated cardiac markers. Plaque inclusion criteria were as follows: (1) plaque located in one of the three major epicardial coronary arteries; (2) angiographic diameter stenosis between 30% and 70% by visual estimation; and (3) availability of easily identified anatomical landmarks both proximally and distally. Plaque exclusion criteria were as follows: (1) poor image quality; (2) severe calcifications; and (3) mismatched images between the different time points. Patients with at least one eligible *de novo* lesion were included. Twenty-nine patients were excluded (5 had no plaques, 8 had no identifiable anatomical landmarks, 7 had poor image quality, and 9 had mismatched images). Finally, 138 patients with 213 plaques were included in this study.

### IVUS imaging and analysis

IVUS images were acquired as previously described [25]. Briefly, aspirin (300 mg), clopidogrel (300 mg), and heparin (100 U/kg) were administered before cardiac catheterization. Cardiac catheterization was performed by radial or femoral approach using a 6-F guiding catheter. IVUS examinations were performed after intracoronary administration of nitroglycerin (100-200 μg) using a 40 MHz Atlantis Pro catheter (Boston Scientific, Boston, MA, USA). The IVUS catheter was pulled back automatically at 0.5 mm/s. For all patients, follow-up imaging was performed using the same IVUS system that was used at baseline.

IVUS image analysis was performed by two independent investigators according to the standards of the American College of Cardiology using EchoPlaque software (Indec Systems, Mountain View, CA, USA) [5]. Baseline IVUS-derived plaque characteristics were evaluated. Attenuated plaques were defined as hypoechoic plaques with ultrasound attenuation in the absence of calcification or dense fibrous tissue. Spotty calcifications were defined as the presence of deposits measuring 1-4 mm in length and containing an arc of calcification < 90°. Plaque burden was calculated as the plaque area divided by the EEM area and multiplied by 100. Distal and proximal references were the sites with the least amount of plaque within 10 mm distal and proximal to the lesion with no major intervening branches. The static remodeling index was determined by comparing lesion site EEM area to the average of the proximal and distal reference EEM area. Static positive remodeling was defined as a remodeling index ≥ 1.05.

Serial IVUS images from baseline and follow-up were analyzed side-by-side, and pullbacks were compared frame-by-frame to identify and match corresponding vessel segments. Identifiable anatomic structures were used as landmarks and included the carina of LAD and LCX, the ostium of RCA, stents, and large side branches. EEM, lumen, and plaque areas were measured at 1-mm intervals in each coronary target segment for each IVUS time point. Mean lumen, plaque, and EEM areas of lesions at each time point and changes from baseline to the 12-month follow-up evaluation were calculated. The slopes (β) of the regression lines relating changes in EEM areas to changes in plaque areas were calculated. We assessed the capacity for vascular remodeling in response to changes in plaque area between different plaque types by comparing the regression line slopes. As to individual plaques, the level of change in EEM area/change in plaque area was used to indicate the vascular remodeling capacity of each plaque [5].

### OCT imaging and analysis

**Figure 3:**
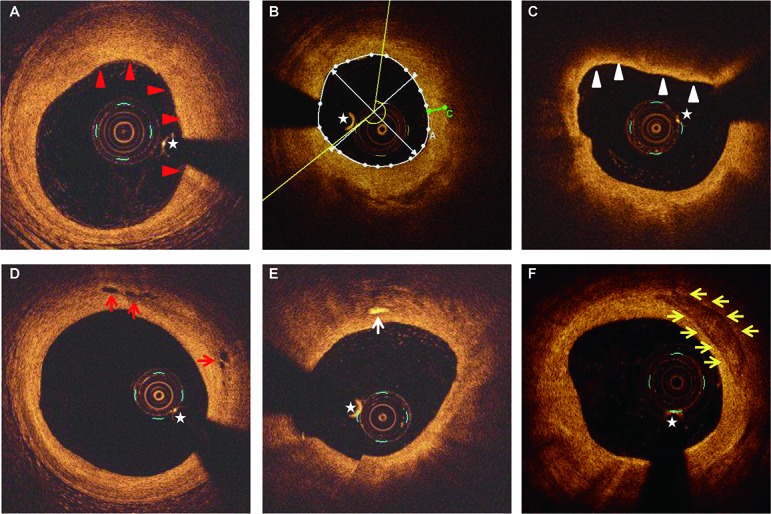
Representative OCT images **A.** Fibrous plaque characterized by a homogeneous signal-rich area (red arrowheads). **B.** Measurement of lipid arc (yellow lines) and fibrous cap thickness (green line) for a lipid plaque. **C.** TCFA is defined by the presence of thin fibrous cap (< 65μm) overlying a lipid-rich plaque (maximum lipid arc > 90°) (white arrowheads). **D.** Microvessels are identified as signal-absent holes within a plaque (red arrows). **E.** Cholesterol crystals are defined as thin, linear regions of high signal intensity within a plaque (white arrowheads). **F.** Calcifications are identified as sharply demarcated areas with low signal attenuation (yellow arrows). Asterisk indicates guide-wire shadowing artifact.

OCT imaging was performed as previously described using a frequency-domain (C7-XR OCT Intravascular Imaging System, St. Jude Medical, St. Paul, MN, USA) or time-domain (M2/M3 Imaging System, LightLab Imaging, Westford, MA, USA) OCT system [25]. OCT images were analyzed by two experienced reviewers with proprietary offline software (LightLab Imaging). Fibrous plaques were identified as homogenous signal-rich areas. Lipid plaques were defined as signal-poor regions with diffuse borders. Lipid was evaluated by measuring lipid length on the longitudinal view and lipid arc on the cross-sectional view every 1-mm interval throughout the lesion. Fibrous cap thickness of lipid plaque was measured thrice at its thinnest part, and then the values were averaged. Thin-cap fibroatheroma (TCFA) was defined as a plaque with > 90° of maximum lipid arc and cap thickness < 65 μm. A microvessel was defined as a sharply delineated signal-poor void with a diameter of 50-300 μm, which was not connected to the vessel lumen and was noted on > 3 consecutive frames [26]. Calcification was characterized as a heterogeneous area with low signal intensity and sharp borders. Macrophage accumulation was defined as increased signal intensity within a lesion, accompanied by high signal attenuation casting a heterogeneous shadow. Representative OCT images are shown in Figure 3.

### Quantitative coronary angiography

Minimum lumen diameter, reference diameter, and diameter stenosis were measured after calibration with the catheter tip using CAAS 5.10.1 software (Pie Medical Imaging BV, Maastricht, Netherlands).

### Statistical analysis

All statistical analyses were performed using SPSS analytical software (version 17.0; SPSS, Inc., Chicago, IL, USA). Categorical variables are presented using frequencies and percentages, and continuous variables are presented with mean and standard deviations or median and interquartile ranges, according to their distribution. Paired t-tests or Wilcoxon tests were applied to analyze within-group differences (baseline vs. follow-up). Spearman's rank correlation coefficient was calculated. To account for the presence of multiple plaques in each patient, the slopes (β) of the regression lines relating changes in EEM and lumen areas to changes in plaque areas were calculated using the generalized estimating equations (GEE). If the slope of the regression line differed significantly from zero in the subgroups, the assumption of homogeneity of regression slopes was further tested by GEE. Multiple linear analyses, using the level of change in EEM area/change in plaque area as the dependent variable, were performed with GEE to identify independent predictors for arterial remodeling capacity. A P-value < 0.05 was considered statistically significant.
